# Correlation of dynamic membrane fluctuations in red blood cells with diabetes mellitus and cardiovascular risks

**DOI:** 10.1038/s41598-021-86528-0

**Published:** 2021-03-26

**Authors:** Minji Sohn, Ji Eun Lee, MinGeun Ahn, YongKeun Park, Soo Lim

**Affiliations:** 1 Department of Internal Medicine, Seoul National University Bundang Hospital, Seoul National University College of Medicine, Seongnam, Republic of Korea; 2 Department of Physics, Korea Advanced Institute of Science and Technology, Daejeon, Republic of Korea; 3 Tomocube Inc., Daejeon, 34051 Republic of Korea

**Keywords:** Biomarkers, Cardiology, Endocrinology

## Abstract

The rheological and physiological properties of red blood cells (RBCs) are affected by many factors in the vascular environment. Among them, membrane fluctuations (MFs), particularly dynamic fluctuations in RBC cell membrane thickness (RBC-MFs), are likely to be altered by the level of glycation of haemoglobin in patients with diabetes mellitus (DM). We investigated the associations of RBC-MFs with physiological variables associated with DM and cardiovascular diseases (CVDs). Forty-one healthy control subjects and 59 patients with DM were enrolled. Five-microliter samples of blood were collected and diluted 400 times. To measure the RBC-MFs, holotomography was used, which non-invasively and precisely analyses the optical characteristics of RBCs. Associations between the RBC-MFs and biochemical parameters related to glucose homeostasis and lipid profiles were investigated. Independent associations of the RBC-MFs with the presence of CVDs were also analysed. RBC-MFs were lower in patients with DM than in healthy participants (61.64 ± 7.49 nm vs 70.65 ± 6.65 nm, *P* = 1.4 × 10^−8^). RBC-MFs correlated modestly with glycated haemoglobin level (*ρ* = − 0.47) and weakly with age (*ρ* = − 0.36), duration of diabetes (*ρ* = − 0.36), fasting plasma glucose level (*ρ* = − 0.37), and the 10-year Framingham risk score (*ρ* = − 0.38) (all *P* < 0.05). Low RBC-MFs were independently associated with the presence of CVDs after adjusting for CVD risk factors. The weak but significant associations of RBC-MFs with cardiometabolic risk factors and CVDs suggest that such deformity of circulating RBCs may be a useful marker of vascular complications of DM.

## Introduction

In subjects with impaired glucose homeostasis or with diabetes mellitus (DM), the rheological and physiological properties of blood and red blood cells (RBCs) are changed^1^. In particular, RBCs can shrink passively from 8 μm down to 4 μm in diameter to pass through small blood vessels. During the progress of atherosclerosis and other vascular diseases, blood vessels become narrowed so that RBCs have to shrink even more^2^. These phenomena are commonly found in patients with DM and vascular complications^3^. Therefore, RBC deformability has been studied to understand the pathophysiology of DM and its complications^4^. Various techniques, such as measuring osmosis, atomic force, microfluidics, and optical characteristics have been developed to detect RBC deformability^4^.

Among experimental techniques to probe the deformability of individual RBCs, holotomography, one of the three-dimensional (3-D) quantitative phase imaging techniques, has been utilised for its quantitative imaging capability^5^. Exploiting laser interferometric imaging and a tomographic reconstruction algorithm, holotomography measures and quantifies both the 3-D refractive index (RI) distributions and dynamic membrane fluctuations (MFs) of individual RBCs. The principle of holotomography is an optical analogy of computed tomography^6^. By measuring multiple two-dimensional (2-D) optical field images of a sample using various illumination angles, a 3-D RI tomogram is retrieved using a reconstruction algorithm. In addition, time-lapse 2-D images of individual RBCs are measured, from which dynamic MFs are calculated. The averaged dynamic fluctuation over the cell membrane is used as an index for RBC deformability. This dynamic MF measure has been utilised in various studies for investigating the biophysical properties of RBC membranes and in addressing RBC deformability with diseases^7,8^.

Advanced holotomography has several advantages over other techniques. Atomic force microscopy (AFM), ektacytometry, and the holographic optical tweezer apply a large force to RBCs, which causes the cells to undergo large deformation, leads to marked changes in cell shape, and makes it difficult to analyse RBC deformability because of nonlinear mechanics such as strain-stiffening^4,9,10^. However, holotomography measures intrinsic membrane undulation and thus provides information about the intrinsic deformability of RBCs^10,11^. The sampling process is simple and can be performed on a finger-prick blood sample. The analysis is also simple, can be completed within seconds, and does not require sophisticated methods such as labelling of RBCs.

At present, the implications of RBC deformability for the pathophysiology of vascular complications differ depending on the methods^12,13^. However, a standard method for analysing RBC deformability has yet to be determined. A previous study analysing optical parameters of RBCs using 3-D holotomography with 12 subjects reported that changes in RBC-MFs were more evident in patients with DM compared with other morphological parameters, such as volume, surface area, and sphericity^14^. However, the exact clinical significance has not yet been well determined. We hypothesised that RBC-MFs, a representative phenotype of RBC deformability, would be related to DM and vascular complications. We analysed the associations of RBC-MFs with physiological variables associated with DM and risk of cardiovascular diseases (CVDs) in healthy subjects and patients with DM. Its associations with DM complications were also investigated.

## Results

### Participants’ characteristics

The demographic and biochemical characteristics of the participants are presented in Table 1. There was a significant difference in anthropometric and biochemical values between the control and study participants except for sex, DBP, total cholesterol and LDL-c concentrations, and haematological values, such as haematocrit, haemoglobin concentration, and RBC, white blood cell, and platelet counts. Some of the healthy control subjects were diagnosed with dyslipidaemia (N = 1, 2%) and being overweight or obese (N = 7, 17%). However, in the patients with DM, the prevalence of hypertension, dyslipidaemia, and CVD were 56% (N = 33), 64% (N = 38), and 39% (N = 23), respectively.

**Table 1:** Participants’ characteristics.

Variable	Healthy subjects (N = 41)	Patients with DM (N = 59)	*P*
Age, year	33.1 ± 3.5	59.6 ± 14.0	2.2 × 10^−16^
Male, n (%)	21 (51.2)	29 (49.2)	1
Body mass index, kg/m^2^	22.5 ± 3.5	26.8 ± 4.40	8.5 × 10^−7^
Smoking history, n (%)	0 (0)	17 (22.8)	4.6 × 10^−4^
Smoking, pack-year	0.0 ± 0.0	6.1 ± 11.8	1.8 × 10^−4^
Waist circumference, cm	80.4 ± 9.9	90.3 ± 11.4	1.9 × 10^−5^
Systolic blood pressure, mmHg	125.5 ± 9.7	136.6 ± 17.7	8.2 × 10^−4^
Diastolic blood pressure, mmHg	74.3 ± 6.3	75.1 ± 10.0	0.654
AST, IU/L	26.2 ± 16.2	36.3 ± 29.5	0.031
ALT, IU/L	19.6 ± 9.9	35.1 ± 26.9	1.1 × 10^−4^
Fasting plasma glucose, mg/dL	94.3 ± 8.1	177.7 ± 64.0	2.7 × 10^−14^
HbA1c, %	5.3 ± 0.3	9.2 ± 1.3	2.2 × 10^−16^
Total cholesterol, mg/dL	183.2 ± 32.7	170.9 ± 42.3	0.119
Triglycerides, mg/dL	109.3 ± 74.5	152.7 ± 112.0	0.022
LDL-cholesterol, mg/dL	108.6 ± 24.8	101.1 ± 31.7	0.208
HDL-cholesterol, mg/dL	59.2 ± 12.9	50.3 ± 11.2	3.8 × 10^−4^
eGFR, mL/min/1.73 m^2^	114.6 ± 11.4	93.2 ± 21.0	3.4 × 10^−9^
Urinary protein-to-Cr ratio, mg/g*	81.7 ± 99.2	386.7 ± 1289.2	3.1 × 10^−7^
Urinary albumin-to-Cr ratio, mg/g*	19.7 ± 78.6	219.6 ± 935.0	1.2 × 10^−6^
Haemoglobin, g/dL	14.2 ± 1.6	14.1 ± 1.8	0.872
RBC, 10^6^/μL	4.7 ± 0.5	4.8 ± 0.6	0.975
Hematocrit, %	42.7 ± 4.2	42.8 ± 4.7	0.907
WBC, 10^3^/μL	6.6 ± 1.8	7.1 ± 2.1	0.270
Platelet, 10^3^/μL	269.7 ± 59.6	244.9 ± 67.1	0.063
**Comorbidity**			
Hypertension, n (%)	–	33 (55.9)	–
Dyslipidaemia, n (%)	1 (2.4)	38 (64.4)	1.5 × 10^−9^
Obesity, n (%)	7 (17.1)	36 (61.0)	3.2 × 10^−5^
Diabetic nephropathy, n (%)	–	23 (39.0)	–
Diabetic neuropathy, n (%)	–	5 (8.5)	–
Diabetic retinopathy, n (%)	–	13 (22.0)	–
Cardiovascular diseases, n (%)	–	23 (39.0)	–
**Concomitant medication**			
Antihypertensive agents, n (%)	–	31 (60.8)	–
Lipid-lowering agents, n (%)	–	31 (60.8)	–
Antiplatelet agents, n (%)	–	19 (32.2)	–

Values are expressed as the mean ± SD or number (%). Smoking history refers to current or ex-smoker. AST, aspartate aminotransferase; ALT, alanine aminotransferase; LDL, low-density lipoprotein; HDL, high-density lipoprotein; eGFR, estimated glomerular filtration ratio; Cr, creatinine; RBC, red blood cell; WBC, white blood cell.

*Values compared after logarithmic transformation.

### Association of the RBC-MFs with biochemical parameters and clinical phenotypes

**Figure 1:**
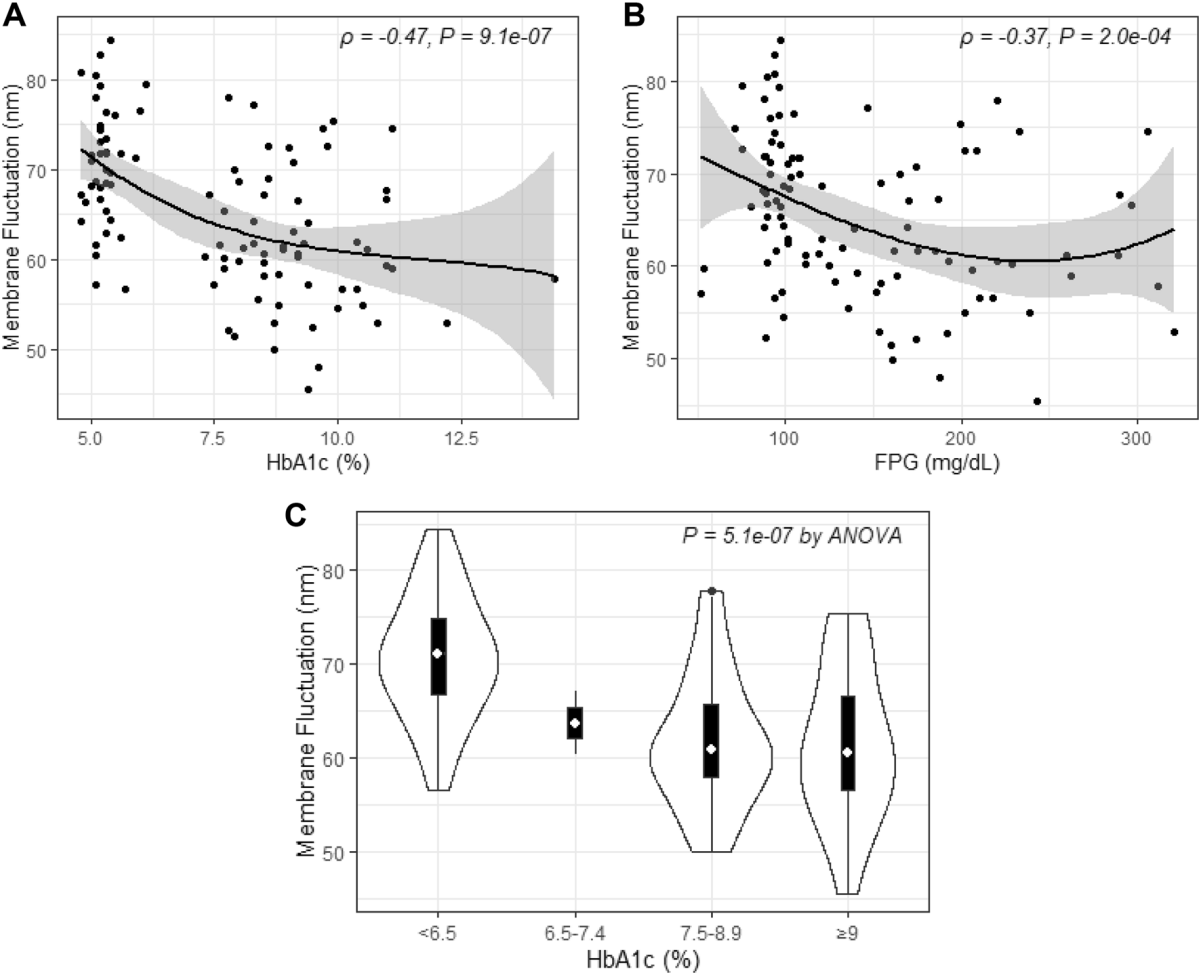
Correlations between the membrane fluctuations of red blood cells and clinical factors. (**A**) HbA1c (%), (**B**) FPG, fasting plasma glucose (mg/dL), (**C**) Groups divided by HbA1c levels as follows: < 6.5%, 6.5%–7.4%, 7.5%–8.9%, and ≥ 9%.

Negative correlations were found between RBC-MFs and HbA1c level (*ρ* = − 0.47, *P* = 9.1 × 10^−7^; Fig. 1A) and between RBC-MFs and fasting plasma glucose (FPG) concentration (*ρ* = − 0.37, *P* = 2.0 × 10^−4^; Fig. 1B). For HbA1c levels, the RBC-MF values were 70.65 ± 6.65 nm in the < 6.5% group, 63.70 ± 4.81 nm in the 6.5–7.4% group, 61.73 ± 7.21 nm in the 7.5–8.9% group, and 61.41 ± 8.07 nm in the ≥ 9% group (Fig. 1C).

The RBC-MF values according to clinical status are presented in Table 2. RBC-MFs were significantly lower by 9.01 ± 1.42 nm in patients with DM compared with controls. RBC-MFs were also significantly lower in patients with CVD, diabetic nephropathy, diabetic neuropathy, hypertension, or dyslipidaemia.

**Table 2:** Membrane fluctuations of red blood cells associated with clinical status in all participants.

Variable	Yes	No	*P*
Male (N = 50)	65.01 ± 8.72	65.66 ± 8.15	0.700
Smoking history (N = 17)	60.95 ± 10.04	66.23 ± 7.79	0.017
Drinking history (N = 11)	62.43 ± 10.54	65.69 ± 8.10	0.226
**Comorbidity**			
Diabetes mellitus (N = 59)	61.64 ± 7.49	70.65 ± 6.65	1.4 × 10^−8^
Diabetic nephropathy (N = 23)	61.53 ± 8.84	66.47 ± 7.98	0.013
Diabetic neuropathy (N = 5)	57.08 ± 5.66	65.77 ± 8.32	0.023
Diabetic retinopathy (N = 12)	63.35 ± 9.74	65.63 ± 8.21	0.230
Hypertension (N = 33)	61.35 ± 7.63	67.29 ± 8.11	6.7 × 10^−4^
Dyslipidaemia (N = 39)	61.80 ± 7.82	67.59 ± 8.03	5.8 × 10^−4^
Obesity (N = 43)	63.83 ± 8.56	66.47 ± 8.17	0.120
Cardiovascular diseases (N = 23)	59.76 ± 8.34	67.00 ± 7.72	1.9 × 10^−4^

Values are expressed as the mean ± SD of membrane fluctuation (nm). Smoking history refers to current or ex-smokers. Drinking history refers to current or ex-drinkers.

The relationships between RBC-MF values and clinical factors are presented in Supplementary Tables S1 and S2. The RBC-MF values were weakly negatively correlated with age (*ρ* = − 0.36, *P* = 4.1 × 10^−4^). RBC-MF values did not correlate significantly with any haematological values.

### Association of RBC-MF values with cardiovascular (CV) risks

**Figure 2:**
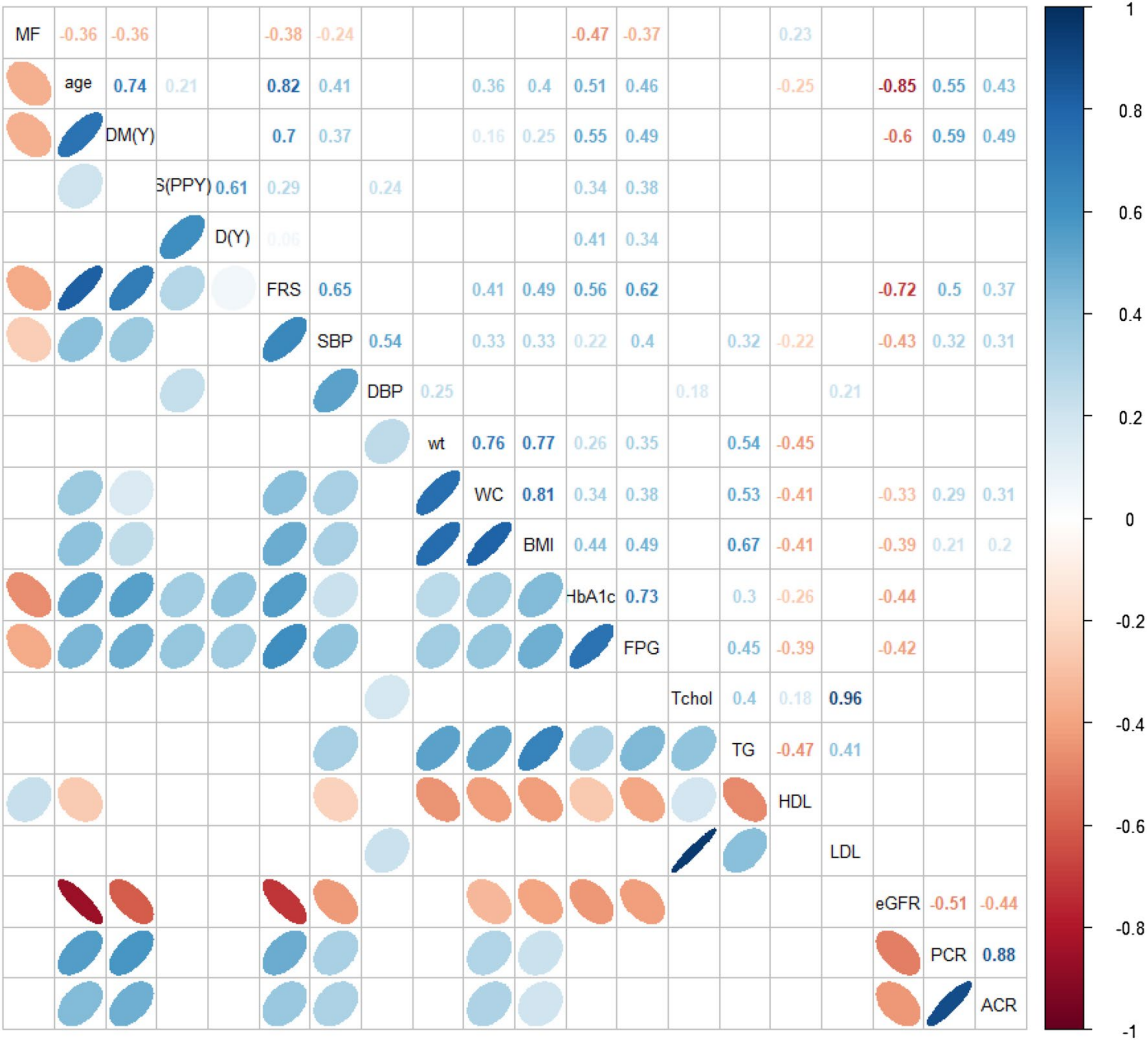
Correlations between cardiovascular risks and the membrane fluctuation of red blood cells. The upper part of the figure shows Spearman’s correlation coefficients (*ρ* values) and the lower side of the figure shows correlations by direction and thickness. Only significant correlations (*P* < 0.05) are retained in the picture. MF, membrane fluctuations; DM(Y), duration of diabetes mellitus (year); S(PPY), smoking duration (packs per year); D(Y) drinking duration (years); FRS, 10-year coronary heart disease risk by Framingham risk score; SBP, systolic blood pressure; DBP, diastolic blood pressure; wt, weight; WC, waist circumference; BMI, body mass index; HbA1c, glycated haemoglobin; FPG, fasting plasma glucose; Tchol, total cholesterol; TG, triglycerides; HDL, high-density lipoprotein cholesterol; LDL, low-density lipoprotein cholesterol; eGFR, estimated glomerular filtration ratio; PCR, urinary protein-to-creatinine ratio; ACR, urinary albumin-to-creatinine ratio.

The relationships between CV risk factors and RBC-MFs are shown in Fig. 2 and Supplementary Table S2. RBC-MFs correlated weakly negatively with the duration of DM (*ρ* = − 0.36, *P* = 4.2 × 10^−4^). RBC-MFs correlated negatively with SBP (*ρ* = − 0.24, *P* = 0.017), but this correlation was even weaker.

**Figure 3:**
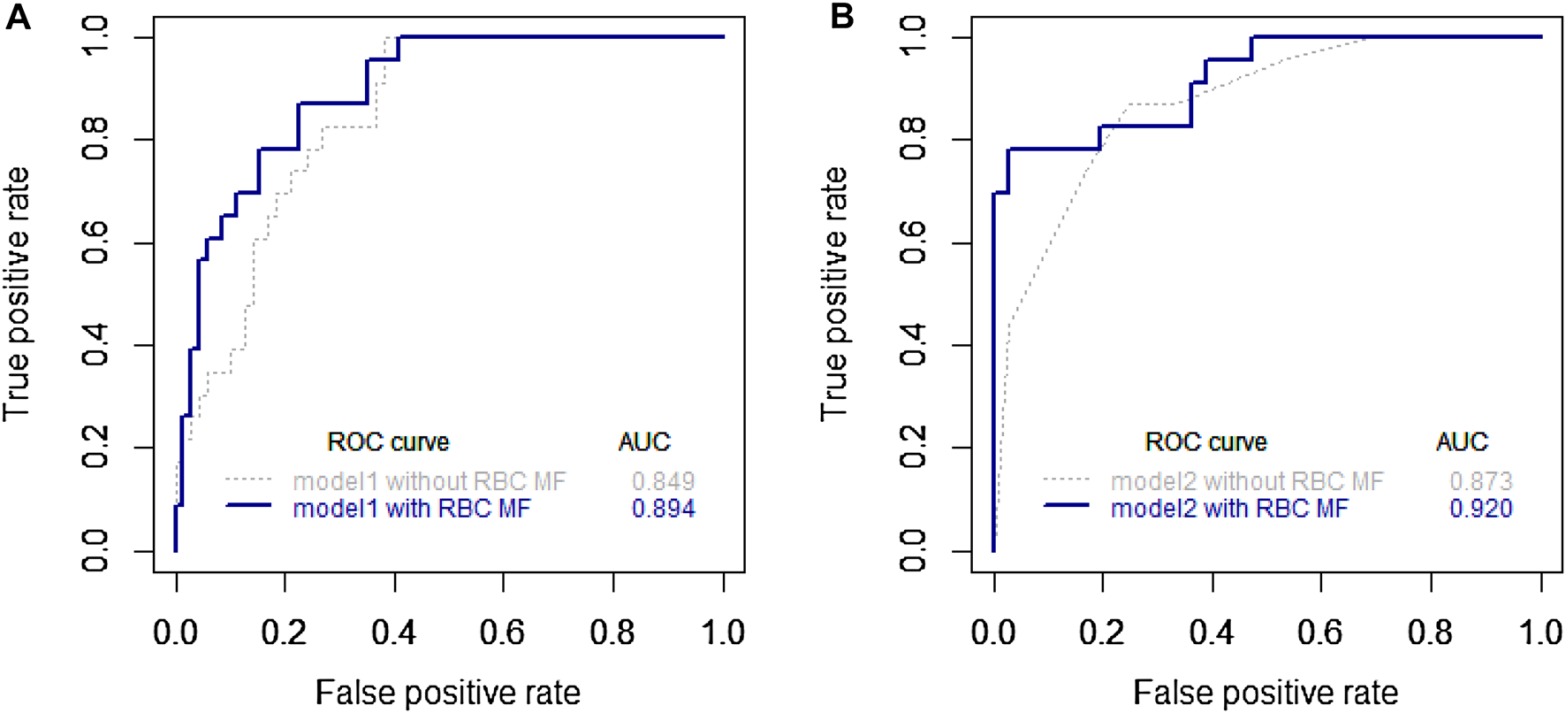
Receiver-operating-characteristic (ROC) curves for CVD by multivariable logistic regression models with or without red blood cell membrane fluctuations (RBC-MFs). (**A**) *Model 1* tested in all participants; (**B**) *Model 2* tested in patients with diabetes mellitus (DM).

The results of logistic regression analysis for CVD are shown in Table 3. For multivariable analysis in all subjects, RBC-MFs, HbA1c, and log PCR remained as independent risk factors in *Model 1*. In a multivariable analysis for patients with DM, RBC-MFs, hypertension, dyslipidaemia, and chronic kidney disease remained as independent risk factors (*Model 2)*. The AUC was increased when RBC-MF values adjusted with hematocrit were added to the models with known risk factors, but the difference was not statistically significant (AUC 0.894 vs 0.849 in *Model 1* and 0.920 vs 0.873 in *Model 2*) (Fig. 3).

**Table 3:** Investigation of Independent Association of Clinical and Biochemical Parameters including Membrane Fluctuations of Red Blood Cells with Cardiovascular Disease.

Variables	Model 1		Variables	Model 2	
	Crude odds ratio	Adjusted odds ratio		Crude odds ratio	Adjusted odds ratio
RBC-MFs (nm)	0.98 (0.97–0.99)	0.99 (0.98–1.00)	RBC-MFs (nm)	0.99 (0.97–1.00)	0.99 (0.97–1.00)
Age (year)	1.01 (1.01–1.02)	–	Age (year)	1.01 (1.00–1.02)	–
BMI (kg/m^2^)	1.03 (1.01–1.05)	–	Smoking history	1.13 (0.68–1.86)	–
SBP (mmHg)	1.01 (1.00–1.01)	–	Hypertension	1.42 (1.12–1.81)	1.29 (1.06–1.57)
HbA1c (%)	1.09 (1.06–1.13)	1.05 (1.01–1.10)	Obesity (≥ 25 kg/m^2^)	1.15 (0.89–1.49)	–
LDL-c (mg/dL)	1.00 (1.00–1.00)	–	Dyslipidaemia	1.70 (1.36–2.13)	1.61 (1.31–1.97)
Urinary PCR (mg/g)*	1.24 (1.14–1.35)	1.15 (1.05–1.26)	Chronic kidney disease	1.33 (1.04–1.71)	1.17 (0.96–1.43)

Data are expressed as adjusted odds ratios with 95% confidence intervals. Multivariable logistic regression analyses were performed by backward elimination method. *Model 1* was tested in all participants. *Model 2* was tested in diabetic patients. RBC-MF, red blood cell-membrane fluctuation; SBP, systolic blood pressure; LDL-c, low-density lipoprotein cholesterol; PCR, protein-to-creatinine ratio; BMI, body mass index.

*Logarithmic-transformed value was used.

**Figure 4:**
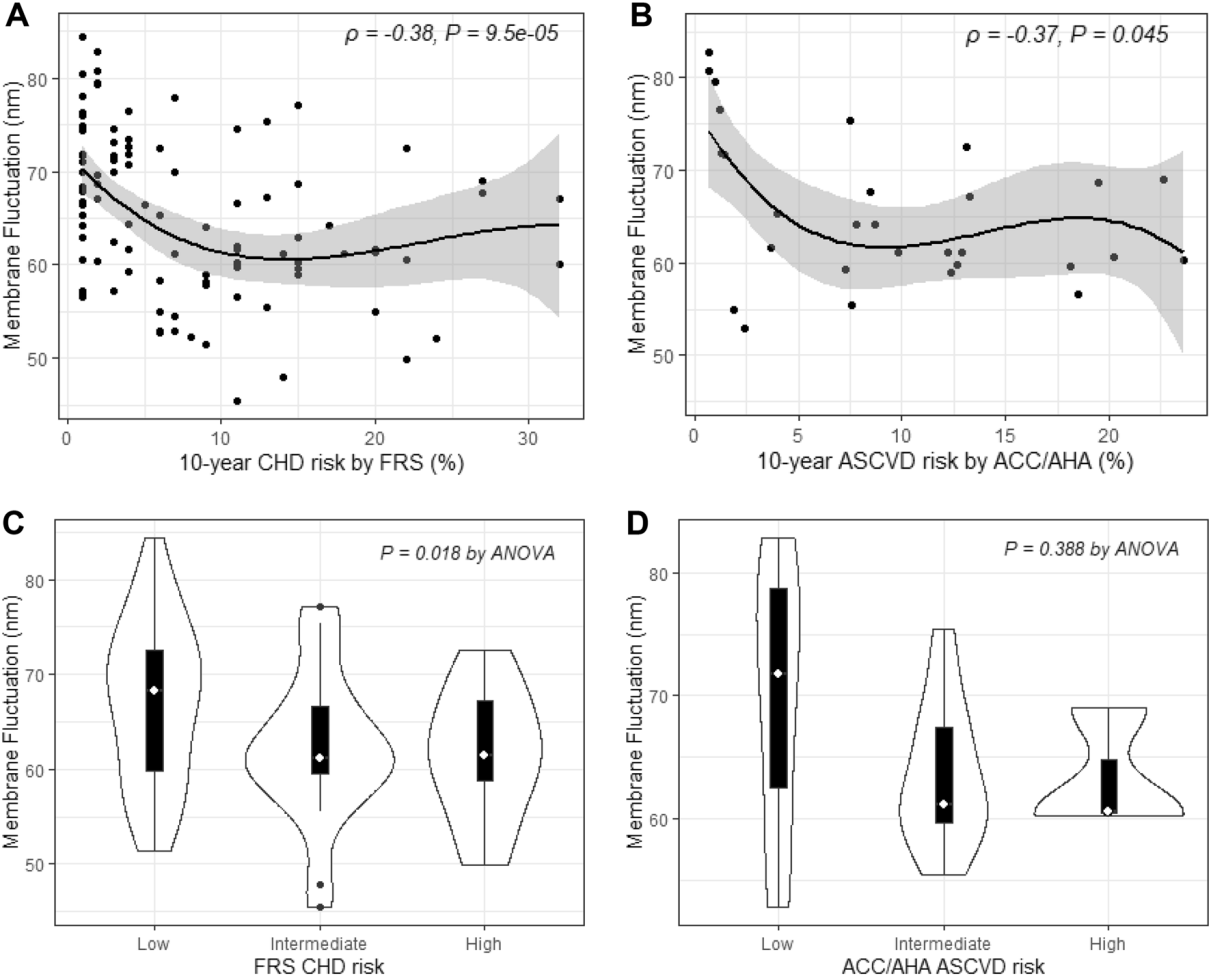
Correlation between red blood cell-membrane fluctuations (RBC-MFs) and 10-year cardiovascular disease (CVD) risks. (**A**) Ten-year CHD risk by FRS, (**B**) Ten-year ASCVD risks by ACC/AHA guidelines, (**C**) Ten-year CHD risk with risk criteria as follows: low (< 10%), intermediate (10–19%), and high (≥ 20%), (**D**) 10-year ASCVD risk with risk criteria as follows: low (< 5%), borderline (5–7.4%), intermediate (7.5–19.9%), and high (≥ 20%). CHD, coronary heart disease; FRS, Framingham risk score; ASCVD, atherosclerotic CVD; ACC, American College of Cardiology; AHA, American Heart Association.

Correlations between RBC-MFs and 10-year CV risks are shown in Fig. 4. Weak negative correlations were found between RBC-MFs and 10-year coronary heart disease (CHD) risk by Framingham risk score (FRS) (*ρ* = − 0.38, *P* = 9.5 × 10^−5^), and between RBC-MFs and 10-year atherosclerotic CVD (ASCVD) risk using the American College of Cardiology/American Heart Association (ACC/AHA) criteria (*ρ* = − 0.37, *P* = 0.045). The mean ± SD of RBC-MFs was 66.98 ± 8.42 nm in the low-risk, 62.24 ± 7.85 nm in the intermediate-risk, and 61.55 ± 6.85 nm in the high-risk groups classified using the 10-year CHD risk estimated using FRS. According to the 10-year ASCVD risk using ACC/AHA criteria, the RBC-MF values were 69.79 ± 10.70 nm in the low-risk, 63.57 ± 5.74 nm in the intermediate-risk, and 63.30 ± 4.94 nm in the high-risk groups.

### Association of other characteristics of RBCs with clinical phenotypes

Along with MFs, the holotomography system used in this study provides four morphological characteristics of RBCs: cell volume (fL), surface area (μm^2^), sphericity index, and haemoglobin protein density (g/dL). The comparison of these characteristics of RBCs between healthy subjects and patients with DM is presented in Fig. S1. The cell volume and surface area were significantly greater in patients with DM than in healthy subjects.

RBC surface area correlated moderately with RBC-MFs (*ρ* = − 0.45, *P* = 3.6 × 10^−5^; Fig. S2) and was significantly higher in patients with DM and other complications (Supplementary Table S2). We conducted a similar logistic regression analysis of the relationship between CVD and the surface area of RBCs but found no independent association between these variables (Supplementary Table S3).

## Discussion

In this cross-sectional pilot study, we compared healthy control subjects and patients with DM. We found that the RBC-MF value was lower in patients with DM than in healthy subjects. RBC-MFs correlated negatively with clinical parameters including age, HbA1c and FPG levels, and SBP. In addition, the RBC-MFs were independently associated with the presence of CVD, even after adjusting for known CV risk factors.

The RBC-MF values in this study were similar to those in a previous study^14^ and the magnitude of difference between healthy subjects and patients with DM was similar. These results are supported by reports that DM affects the RBC membrane, which can be damaged by lipid peroxidation end-products^15^. RBC aggregability changes with changes in membrane phospholipid levels in patients with DM^16^. HbA1c level is linked to RBC membrane glycosylation^17^. In addition, hyperglycaemia is related to reduced solubility and mobility of RBCs because of an increased content of β-sheet structure^18^.

The RBC-MF values were correlated with future CV risk scores estimated by the FRS and ACC/AHA 10-year risk criteria. They were also associated with the presence of CVDs independently of other CV risk factors. The pathophysiological roles of RBC-MFs in CVDs are unknown. Blood viscosity, fluidics, RBC distribution width (RDW), and RBC oxidative stress were measured to investigate associations with vascular health^19,20^. As for the RBC membrane, transport systems, such as Na^+^-K^+^ ATPase, are altered in patients with hypertension^20^. It can be assumed that circulating RBCs with decreased MFs indicate the hardness of RBCs, which increases stresses to the blood vessels. How RBC-MFs affect CVDs can be explained by the lipid composition of RBC membranes. The RBC membrane is composed of 43% lipid molecules and a quarter of them are cholesterol, which is 1.5 to 2.0 times richer than in other cells^21,22^. Focusing on the positive correlation of RBC-MF values with HDL-c concentration, this molecule might influence the RBC membrane with antiapoptotic and anti-inflammatory effects and help improve the environment of blood vessels particularly for endothelial cells^23,24^. Thus, given that the structure and function of RBCs are affected by the vascular environment, RBC-MFs are likely to be decreased in poor vascular conditions, such as CVDs. Further mechanistic studies are warranted to identify the role of RBC-MFs in vascular health and disease.

Here, subjects with nephropathy and neuropathy had lower RBC-MFs than those without these conditions. Nephropathy is a representative microvascular complication of DM; it is associated with vascular stiffness and is affected by the renal microenvironment^25^. The kidneys are a source of erythropoietin, which has its primary effect on RBC progenitors and precursors by promoting their survival by protecting them from apoptosis or cell death^26^. Thus, functional aspects of RBCs can be altered in DM nephropathy. Regarding this, only four patients had an eGFR lower than 60 mL/min/1.73m^2^ given our inclusion criteria. Thus, more patients with moderate to severe kidney dysfunction are required to evaluate the relationship of RBC-MFs with more severe renal complications.

The links between RBC-MFs, age, and smoking habits in our results may be explained by previous findings. RBC morphology and biophysical properties change with age, which might arise from cumulative oxidative stress, loss of lipid asymmetry, and reduced enzyme activities, including transaminases^27^. Decreases in RBC-MFs in subjects with a history of smoking can be explained by the roughening of RBC membranes caused by the damaged lipid bilayer^28^.

Although some studies have explored the relationship between DM and RBC deformability, few have examined the relationship between these factors and CVDs^29,30^. AFM has been used to study alterations in RBC morphology in patients with DM and to measure fibrinogen-erythrocyte binding forces in RBCs in patients with CVD^30,31^. However, this binding force is not directly related to RBC deformability, and the procedure requires attachment of fibrinogen to the tips used in AFM. The elongation index (EI), as analysed by ektacytometry, was reported to be lower in people with DM and its complications^29,32^. In consideration of the role of elevated RDW in CV events, the relationship between the EI and RDW was analysed to determine whether there was an indirect association, but this relationship was weak.

Our study also had several limitations. First, it was not possible to establish a causal relationship because correlations of the RBC-MFs with many variables were analysed using cross-sectional data. Second, the weak correlations make it difficult to draw definitive conclusions about the relationships between RBC-MFs and clinical parameters. Larger longitudinal studies with specific patient groups are required to evaluate these relationships further.

In conclusion, we found that RBC-MF values can be measured precisely using advanced holotomography with 3-D quantitative phase imaging technology. The RBC-MF values correlated negatively with age, duration of diabetes, Framingham risk score, and fasting glucose and HbA1c levels (all *ρ* > 0.35). RBC-MFs were significantly lower in subjects with a smoking history, DM, dyslipidaemia, and CVD compared with those without these conditions. In the adjusted models, RBC-MF values were significantly associated with the presence of CVD. RBC-MF values are an indicator of the integrity and viability of RBCs, and decreased RBC-MF levels may be a useful biomarker for assessing the complications of DM, including CVD.

## Methods

### Study design and subjects

This was designed as a cross-sectional pilot study. Forty-one healthy subjects and 59 patients with DM aged 20–90 years without pregnancy, malignancy, or other severe organ disorders, such as liver, kidney, and lung diseases, were enrolled.

The study protocol was approved by our independent Ethics Committee/Institutional Review Board (SNUBH: B-1711/435-303). This study complied with the Declaration of Helsinki’s protocol and principles 2013, and followed the International Conference on Harmonization Harmonized Tripartite Guidelines for Good Clinical Practice. The participants volunteered for the study with written informed consent.

### Data collection and sample analysis

Height and body weight were measured using standard methods with the subjects in light clothing^33^. The body mass index (BMI) was calculated as weight (in kilograms) divided by height (in meters) squared. Blood pressure was measured using an automated blood pressure device (Easy X800; Jawon, Seoul, South Korea). Each participant rested for at least 5 min by sitting in a chair with both feet flat on the floor and both arms supported at the level of the heart before the measurement^34^.

Blood and urine samples were taken after 10 h of overnight fasting, for biochemistry assays performed as reported previously^33^. Plasma glucose concentration was measured using a glucose oxidase method (747 Clinical Chemistry Analyzer; Hitachi, Tokyo, Japan). Glycated haemoglobin (HbA1c) levels were measured using a Bio-Rad Variant II Turbo High-Performance Liquid Chromatography Analyzer (Bio-Rad, Hercules, CA, USA) in a National Glycohemoglobin Standardization Program level II certified laboratory. Total cholesterol, triglyceride, high-density lipoprotein cholesterol (HDL-c), and low-density lipoprotein cholesterol (LDL-c) levels were measured using a 747 Clinical Chemistry Analyzer (Hitachi). Urinary albumin was measured using turbidimetry (502X; A&T, Tokyo, Japan), and urinary creatinine was measured using the Jaffe method (Hitachi 7170; Hitachi). The urinary protein- or an albumin-to-creatinine ratio (mg/g) was used for reporting proteinuria or albuminuria, respectively. Complete blood counts included counts of white blood cells, RBCs, haemoglobin, haematocrit, and platelets. Blood samples were collected into EDTA tubes and analysed using a Sysmex XE-2100 instrument (TOA Medical Electronics Co., Kobe, Japan).

Each participant’s medical history and any comorbid diseases were reviewed, and their smoking and drinking habits were recorded. Any disease presence was defined as patients taking prescribed medicines or showing uncontrolled clinical values when RBC-MFs were measured. The uncontrolled clinical values followed standard criteria: DM (HbA1c ≥ 6.5%), hypertension (systolic blood pressure, SBP ≥ 140 mmHg and/or diastolic blood pressure, DBP ≥ 90 mmHg and SBP ≥ 130 mmHg and/or DBP ≥ 80 mmHg for patients with CVD)^35^, dyslipidaemia (LDL-c ≥ 70 mg/dL for patients with CVD, LDL-c ≥ 100 mg/dL for patients with DM, and LDL-c ≥ 160 mg/dL for healthy subjects)^36^, and obesity (BMI ≥ 25 kg/m^2^)^37^. Diabetic nephropathy was defined from the eGFR < 60 mL/min/1.73 m^2^ and/or the urinary albumin-to-creatinine ratio ≥ 30 mg/g)^38^. Diabetic retinopathy included proliferative diabetic retinopathy and nonproliferative diabetic retinopathy. CVDs included CHD, cerebrovascular disease, and peripheral artery disease^38,39^. The FRS (10-year CHD risk by FRS) and 10-year ACC/AHA atherosclerotic CVD risk assessment tool (10-year ASCVD risk by ACC/AHA) were used to calculate the future 10-year CVD risks^39,40^. Because of the age limitation of the calculator, the 10-year ASCVD risk by ACC/AHA was calculated for participants aged 40–79 years.

### Holotomography

**Figure 5:**
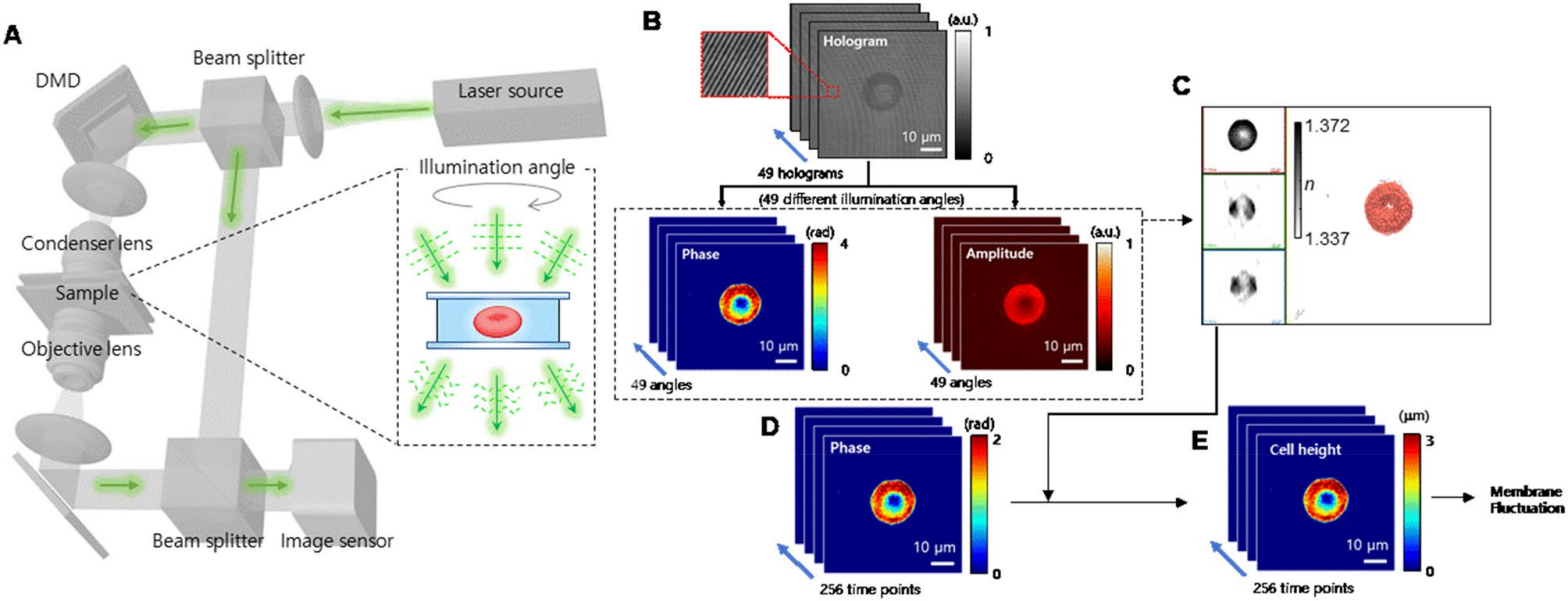
Workflow of the HT system for analysing holotomographic results. (**A**) depicts the used HT system. (**B**–**E**) shows analysis procedure. (**B**) From the multiple 2D hologram patterns of a sample obtained with various illumination angles, both the phase and amplitude images were retrieved. (**C**) From these phase and amplitude images, a 3D RI tomogram is reconstructed. (**D**) Also, the time-lapse phase images of the RBC were recorded with normal illumination. (**E**) Then, from the measure phase images, the time-lapse cell height images were calculated by considering the mean RI values obtained in (**C**).

To measure dynamic MFs of RBCs, a commercial holotomography system (HT-1S; Tomocube Inc., Daejeon, Republic of Korea) was used. The schematic of the used system is presented in Fig. 5A. A diode-pumped solid-state laser (wavelength, *λ* = 532 nm) was used as an illumination source. The beam from the laser source is split into two arms: one for the sample and the other for the reference arm. The sample beam is reflected from a digital micromirror device (DMD), and the angle of the beam impinging onto a sample is controlled systematically by projecting hologram patterns on the DMD^41^. The beam diffracted from a sample is projected onto an image plane, where the sample beam interferes with the reference beam, and generates hologram patterns (Fig. 5B). From the multiple 2-D holograms measured at various illumination angles (N = 49), the phase and amplitude information were retrieved using a phase retrieval algorithm. Then, the 3-D RI tomogram of a cell, *n*_RBC_(*x*, *y*, *z*) was reconstructed using an algorithm of optical diffraction tomography (Fig. 5C)^42,43^. The time-lapse 2-D holograms were measured at a frame rate of 100 Hz (Fig. 5D).

From the time-lapse 2-D holograms, the optical phase delay images Δ*ϕ*(*x*, *y*, *t*) of an RBC were measured for 256 time-points with a frame rate of 150/s. Then, the dynamic cell height images were calculated as Δ*h*(*x*, *y*, *t*) = Δ*ϕ*(*x*, *y*, *t*) ⋅ *λ*/(〈*n*_RBC_〉 − *n*_*m*_)/2π, where 〈*n*_RBC_〉 is the mean RI of an RBC, and *n*_*m*_ is the RI of a given medium (Fig. 5E)^44^. Then, the dynamic RBC-MFs were calculated as the root-mean-square of height difference, Σ[Δ*h*(*x*, *y*, *t*) − 〈Δ*h*(*x*, *y*, *t*)〉]^2^/*N*_*frame*_, where *N*_*frame*_ is the total number of frames. The detailed procedures can also be found elsewhere^45,46^.

### Optical measurement of RBCs

For optical measurement, finger-prick blood samples (5 μL) were collected into cryogenic tubes and the blood was immediately diluted with 2 mL of Dulbecco’s phosphate-buffered saline without calcium or magnesium (10,010,023; Thermo Fisher, Waltham, MA, USA), giving a final dilution of 400 × . The diluted blood was sandwiched between two glass coverslips measuring 25 mm × 50 mm (C025501; Matsunami Glass Ind., Ltd., Osaka, Japan), and then processed using microscopic optical diffusion tomography. The 3-D RI tomograms were obtained using a holotomographic microscope (HT-1S; Tomocube Inc.), and commercial software (Tomostudio; Tomocube Inc.) was used for visualising the measured 3-D RI tomograms. MFs were calculated by continuously recording 2-D-phase RI images using a high-speed camera. Thirty erythrocytes were randomly examined per patient to obtain the average MF index. The same sample was also analysed using an automated hematology analyzer system (XN-1000; Sysmex, Kobe, Japan) for retrieving RBC parameters.

### Statistical methods

Continuous variables are shown as the mean ± standard deviation (SD), and categorical variables are shown as the number and percentage of subjects. Student’s *t* test was used for comparisons for continuous variables and chi-square tests for categorical variables. Spearman’s correlation coefficient (*ρ*) and multivariable logistic regression analyses with 95% confidence intervals (CIs) were used to identify relationships between RBC-MFs and clinical factors. The correlation coefficients were interpreted as previously reported as follows: < 0.1, negligible; ≤ 0.1 to < 0.4, weak; ≤ 0.4 to < 0.7, moderate; ≤ 0.7 to < 0.9, strong; and ≥ 0.9, very strong^47^. Continuous values are preferred in logistic regression analysis but categorical variables were necessary to take account of the effect of medicines related to known risk factors^48^. We tested both continuous and categorical variables. Significant variables determined by univariate logistic regression analysis were used for multivariable analysis. This model was selected by a backward elimination method from the greatest area under the receiver operating characteristic (ROC) curve. The area under the ROC curve (AUC) was compared with or without RBC-MF data to determine its effectiveness. Because the concentration of RBCs is reported to be negatively related to the level of RBC-MF^14,49^, we further adjusted the regression model using hematocrit values. The final multivariable logistic regression analysis model was tested using the Hosmer–Lemeshow method. Statistical significance was assumed at *P* < 0.05. All analyses were performed using R software (version 4.0.2; R Development Core Team, Vienna, Austria) and RStudio (version 1.3.1056; RStudio, Inc., Boston, MA, USA).

## Supplementary Information

Supplementary Information.
