# Author Correction: Correlation of dynamic membrane fluctuations in red blood cells with diabetes mellitus and cardiovascular risks

**DOI:** 10.1038/s41598-021-94538-1

**Published:** 2021-07-27

**Authors:** Minji Sohn, Ji Eun Lee, MinGeun Ahn, YongKeun Park, Soo Lim

**Affiliations:** 1 Department of Internal Medicine, Seoul National University Bundang Hospital, Seoul National University College of Medicine, Seongnam, Republic of Korea; 2 Department of Physics, Korea Advanced Institute of Science and Technology, Daejeon, Republic of Korea; 3 Tomocube Inc., Daejeon, 34051 Republic of Korea

Correction to: *Scientific Reports* 10.1038/s41598-021-86528-0, published online 26 March 2021

The Funding section in the original version of this Article was incomplete.

“This research was funded by Korea Advanced Institute of Science and Technology (Daejeon, Republic of Korea) and SNUBH (Seongnam, Republic of Korea). The funding agency had no role in the study design, data collection and analysis, decision to publish, or preparation of the manuscript.”

now reads:

“This research was funded by Korea Advanced Institute of Science and Technology (Daejeon, Republic of Korea) and SNUBH 16-2015-004 (Seongnam, Republic of Korea). The funding agency had no role in the study design, data collection and analysis, decision to publish, or preparation of the manuscript.”

The original Article has been corrected.

