# Supplementary material for: Correlation of dynamic membrane fluctuations in red blood cells with diabetes mellitus and cardiovascular risks
Source: Sci Rep. 2021 Mar 26;11:7007. doi: 10.1038/s41598-021-86528-0 (PMC7997877; doi:10.1038/s41598-021-86528-0)
Supplement: Supplementary file 1 — Supplementary Information. [file 41598_2021_86528_MOESM1_ESM.docx]

**Supplementary Information**

**Correlation of dynamic membrane fluctuations in red blood cells with diabetes mellitus and cardiovascular risk**

Minji Sohn^1^, Ji Eun Lee^1^, MinGeun Ahn^1^, YongKeun Park^2,3^, and Soo Lim^1^

^1^Department of Internal Medicine, Seoul National University Bundang Hospital, Seoul National University College of Medicine, Seongnam, Republic of Korea

^2^Department of Physics, Korea Advanced Institute of Science and Technology, Daejeon, Republic of Korea

^3^Tomocube Inc., Daejeon, 34051, Republic of Korea.

Corresponding author: Soo Lim, MD, PhD

Department of Internal Medicine, Seoul National University Bundang Hospital, Seoul National University College of Medicine, Seongnam, Republic of Korea

[limsoo@snu.ac.kr](mailto:limsoo@snu.ac.kr)

| **Table S1. Correlation between membrane fluctuation of red blood cells with clinical and biochemical parameters.** | | | |
| --- | --- | --- | --- |
| ***Clinical factor*** | ***ρ*** | ***P-value*** | |
| Age, year | –0.36 | 4.1 × 10^-4^ | |
| Weight, kg | 0.03 | 0.730 | |
| Body mass index, kg/m^2^ | –0.13 | 0.193 | |
| Serum creatinine, mg/dL | 0.11 | 0.293 | |
| Total protein, g/dL | 0.23 | 0.024 | |
| Albumin, g/dL | 0.10 | 0.343 | |
| Aspartate aminotransferase, IU/L | –0.09 | 0.394 | |
| Alanine aminotransferase, IU/L | –0.08 | 0.412 | |
| RBC, 10^6^/μL | –0.16 | 0.105 | |
| Haemoglobin, g/dL | –0.11 | 0.279 | |
| MCV, fL | 0.13 | 0.216 | |
| MCH, pg/cell | 0.11 | 0.261 | |
| WBC, 10^3^/μL | –0.11 | 0.258 | |
| Platelet, 10^3^/μL | –0.01 | 0.905 | |
| *ρ,* *Spearman*’s correlation coefficient. RBC, red blood cell; MCV, mean corpuscular volume; MCH, mean corpuscular haemoglobin; WBC, white blood cell. | | |  |

| **Table S2. Membrane fluctuation of red blood cells with clinical and biochemical parameters.** | | | | |
| --- | --- | --- | --- | --- |
| ***Clinical factors with criteria*** | **Yes** | **No** | ***P-value*** | |
| Age ≥65 years | 61.95 ± 8.05 | 66.29 ± 8.30 | 0.033 | |
| Waist circumference ≥90 cm in male;  ≥85 cm in female | 64.18 ± 9.20 | 66.14 ± 7.78 | 0.268 | |
| Systolic blood pressure ≥130 mmHg | 62.82 ± 7.35 | 68.25 ± 8.60 | 0.002 | |
| Diastolic blood pressure ≥85 mmHg | 61.30 ± 9.08 | 65.63 ± 8.21 | 0.202 | |
| Fasting plasma glucose ≥100 mg/dL | 62.97 ± 7.70 | 69.18 ± 8.16 | 3.2 × 10^-4^ | |
| Total cholesterol ≥200 mg/dL | 67.29 ± 7.96 | 64.71 ± 8.49 | 0.181 | |
| Triglycerides ≥150 mg/dL | 64.63 ± 8.04 | 65.62 ± 8.58 | 0.587 | |
| LDL-cholesterol ≥160 mg/dL | 60.36 ± 7.58 | 65.59 ± 8.40 | 0.201 | |
| HDL-cholesterol <40 mg/dL in male;  <50 mg/dL in female | 63.64 ± 9.20 | 65.76 ± 8.20 | 0.356 | |
| AST ≥40 IU/L | 63.89 ± 9.55 | 65.67 ± 8.14 | 0.460 | |
| ALT ≥40 IU/L | 63.12 ± 9.21 | 65.85 ± 8.18 | 0.245 | |
| Haemoglobin <13.5 g/dL in male;  <12.0 g/dL in female | 66.53 ± 8.11 | 65.01 ± 8.36 | 0.570 | |
| Urinary protein-to-Cr ratio ≥300 mg/g^*^ | 67.30 ± 9.28 | 65.03 ± 8.34 | 0.455 | |
| Urinary albumin-to-Cr ratio ≥30 mg/g^*^ | 62.41 ± 9.68 | 66.25 ± 7.80 | 0.086 | |
| 10-year CHD risk by FRS ≥10% | 61.99 ± 7.40 | 66.98 ± 8.42 | 0.003 | |
| 10-year ASCVD risk by ACC/AHA ≥5% | 63.31 ± 5.41 | 69.79 ± 10.70 | 0.098 | |
| Values are expressed as the mean ± SD of membrane fluctuation (nm). ^*^Values analyzed after logarithmic transformation. AST, aspartate aminotransferase; ALT, alanine aminotransferase; Cr, creatinine; CHD, coronary heart disease; FRS, Framingham risk score; ASCVD, atherosclerotic cardiovascular disease; ACC, American College of Cardiology; AHA, American Heart Association. | | | |  |

## Association of the morphology of red blood cells with clinical phenotypes

The holotomography system (HT-1S; Tomocube Inc., Daejeon, Republic of Korea) used in this study identifies five morphological characteristics of red blood cells (RBCs) along with membrane fluctuations (MFs). These are cell volume (fL), surface area (μm^2^), sphericity index, haemoglobin (Hb) protein density (g/dL), and Hb content (pg).

The cell volume and surface area are directly obtained from the reconstructed 3-D RI tomogram *n*(*x,y,z*), by thresholding and integrating the voxels with the RI values greater than that of a medium *n_m_*. The Hb concentration [Hb] is retrieved from the mean RI value in the cytoplasm of an RBC. The RI difference between the cytoplasm and the surrounding medium $\left\langle\Delta n \right\rangle$ is linearly proportional to the concentrations of intracellular non-aqueous solutes with a proportionality coefficient - refraction increment, α:^1,2^

$\left\langle\Delta n \right\rangle=\left\langle n\left( x,y,z \right) \right\rangle-n_{m}=\left[ Hb \right]$.

The Hb content is calculated by multiplying the retrieved cell volume and [Hb].

The cell volume (65.97 ± 13.75 fL vs 85.97 ± 13.70 fL, *P* = 6.8 × 10^–8^), surface area (152.44 ± 19.76 μm^2^ vs 184.76 ± 14.96 μm^2^, *P* = 1.3 × 10^–11^), and Hb content (19.04 ± 4.31 pg vs 25.04 ± 4.61 pg, *P* = 1.7 × 10^–7^) were significantly higher in patients with DM than in healthy control subjects (Fig. S1). The larger cell volume in patients with DM was also shown in a previous report, which used atomic force microscopy for the analysis of cell volume.^3^ This phenomenon is likely to be driven by osmosis. Glucose is transported by glucose transport protein 1 (GLUT1) in RBCs, and adenosine triphosphate (ATP) is synthesized following glycolysis.^4^ Chronic hyperglycemia causes a progressive increase in the glycation of Hb, which leads to the increase of osmotic pressure. Accordingly, the RBCs in patients with DM tend to swell because of the high inner osmotic pressure.^5^

The correlations between the RBC-MFs and morphological characteristics are shown in Fig. S2. RBC-MFs correlated with cell volume (*ρ* = –0.37, *P* = 8.0 × 10^–4^), surface area (*ρ* = –0.45, *P* = 3.6 × 10^–5^), and Hb content (*ρ* = –0.35, *P* = 0.001). The high correlation between Hb content obtained from holotomography and that obtained for the complete blood counts (*ρ* = 0.99, *P* = 2.2 × 10^–16^) was similar to that reported in a previous study.^2^ The RBC morphology values according to clinical status are presented in Table S2. The values of cell volume and surface area were significantly higher in patients with DM and/or other complications than in healthy controls.


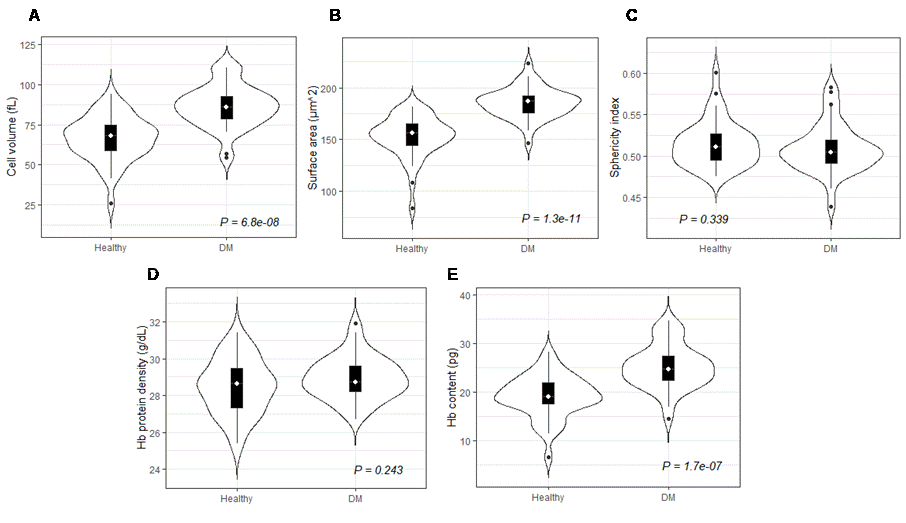


**Figure S1.** Morphological characteristics of red blood cells from healthy subjects and patients with DM. (A) cell volume, (B) surface area, (C) sphericity, (D) Hb protein density, and (E) Hb content. DM, diabetes mellitus; Hb, haemoglobin.

**
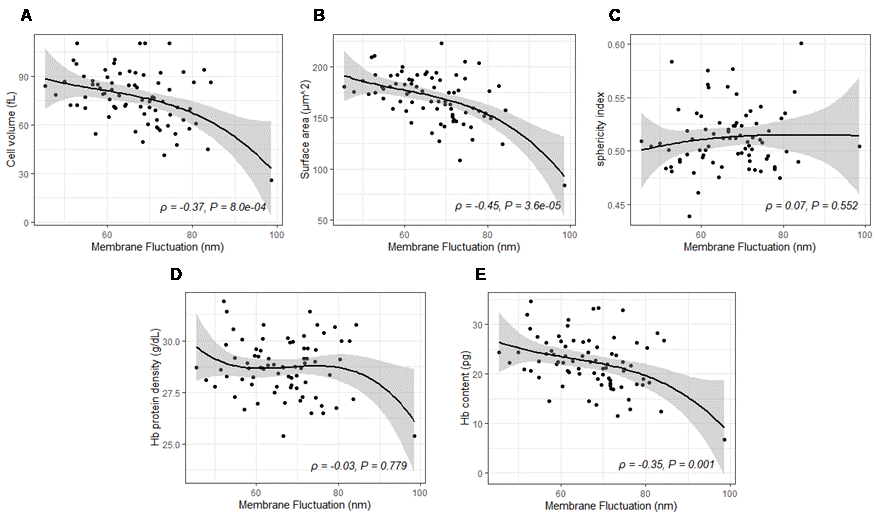
**

**Figure S2.** Correlation between RBC-MFs and morphological characteristics. (A) cell volume, (B) surface area, (C) sphericity, (D) Hb protein density, and (E) Hb content. RBC-MF, red blood cell-membrane fluctuation; Hb, haemoglobin

| **Table S2. Morphological characteristics of red blood cells associated with disease status in all participants** | | | | | | | | | | | | | | | | | | |
| --- | --- | --- | --- | --- | --- | --- | --- | --- | --- | --- | --- | --- | --- | --- | --- | --- | --- | --- |
| **Disease status** | | **Cell volume (fL)** | | ***P*** | | **Surface area (μm^2^)** | ***P*** | **Sphericity index** | ***P*** | | **Hb protein density (g/dL)** | | | ***P*** | | **Hb content**  **(pg)** | | ***P*** |
| Diabetes mellitus | Yes | 85.97 ± 13.70 | 6.8 × 10^-8^ | | 184.76 ± 14.96 | | 1.3 × 10^-11^ | 0.508 ± 0.030 | | 0.339 | | 28.83 ± 1.05 | 0.243 | | 25.04 ± 4.61 | | 1.7 × 10^-7^ | |
|  | No | 65.97 ± 13.75 |  |  | 152.44 ± 19.76 | |  | 0.515 ± 0.028 | |  |  | 28.48 ± 1.49 |  |  | 19.04 ± 4.31 | |  |  |
| *Diabetic nephropathy* | Yes | 84.31 ± 13.32 | 0.015 | | 183.10 ± 13.54 | | 3.7 × 10^-4^ | 0.505 ± 0.028 | | 0.338 | | 28.53 ± 0.97 | 0.637 | | 24.85 ± 4.44 | | 0.012 | |
|  | No | 73.62 ± 17.09 |  |  | 164.30 ± 24.57 | |  | 0.513 ± 0.029 | |  |  | 28.68 ± 1.38 |  |  | 21.50 ± 5.42 | |  |  |
| *Diabetic neuropathy* | Yes | 90.95 ± 3.69 | 0.001 | | 192.59 ± 6.97 | | 0.005 | 0.508 ± 0.008 | | 0.522 | | 28.73 ± 0.82 | 0.880 | | 26.31 ± 1.69 | | 0.023 | |
|  | No | 75.17 ± 16.92 |  |  | 167.11 ± 23.84 | |  | 0.512 ± 0.030 | |  |  | 28.65 ± 1.32 |  |  | 22.12 ± 5.41 | |  |  |
| *Diabetic retinopathy* | Yes | 81.37 ± 16.16 | 0.268 | | 177.28 ± 5.63 | | 0.088 | 0.509 ± 0.036 | | 0.824 | | 28.41 ± 1.13 | 0.501 | | 23.36 ± 5.10 | | 0.491 | |
|  | No | 74.94 ± 16.94 |  |  | 166.71 ± 24.77 | |  | 0.512 ± 0.028 | |  |  | 28.69 ± 1.33 |  |  | 22.12 ± 5.43 | |  |  |
| Hypertension | Yes | 83.17 ± 15.83 | 0.005 | | 179.44 ± 20.75 | | 0.002 | 0.511 ± 0.028 | | 0.848 | | 28.64 ± 1.09 | 0.968 | | 24.55 ± 4.96 | | 0.002 | |
|  | No | 71.75 ± 16.18 |  |  | 161.91 ± 23.44 | |  | 0.512 ± 0.030 | |  |  | 28.65 ± 1.42 |  |  | 20.83 ± 5.16 | |  |  |
| Dyslipidaemia | Yes | 83.99 ± 14.41 | 0.003 | | 182.19 ± 16.21 | | 4.7 × 10^-5^ | 0.507 ± 0.027 | | 0.273 | | 28.64 ± 1.05 | 0.967 | | 24.69 ± 4.76 | | 0.003 | |
|  | No | 71.81 ± 16.67 |  |  | 161.29 ± 24.20 | |  | 0.514 ± 0.030 | |  |  | 28.65 ± 1.42 |  |  | 20.98 ± 5.27 | |  |  |
| Obesity | Yes | 82.34 ± 16.87 | 0.008 | | 177.38 ± 24.33 | | 0.008 | 0.513 ± 0.029 | | 0.769 | | 28.98 ± 1.12 | 0.067 | | 24.29 ± 5.08 | | 0.005 | |
|  | No | 71.59 ± 15.66 |  |  | 162.08 ± 21.85 | |  | 0.511 ± 0.029 | |  |  | 28.43 ± 1.38 |  |  | 20.86 ± 5.16 | |  |  |
| Cardiovascular disease | Yes | 81.38 ± 13.44 | 0.091 | | 179.14 ± 13.20 | | 0.004 | 0.505 ± 0.030 | | 0.307 | | 28.56 ± 1.13 | 0.727 | | 24.13 ± 4.60 | | 0.063 | |
|  | No | 74.26 ± 17.50 |  |  | 165.08 ± 25.39 | |  | 0.514 ± 0.029 | |  |  | 28.68 ± 1.36 |  |  | 21.68 ± 5.50 | |  |  |

| **Table S3. Investigation of independent association of clinical and biochemical parameters including surface area of RBC with CVD** | | | | | |
| --- | --- | --- | --- | --- | --- |
| ***Variables*** | ***Model 1*** | | ***Variables*** | ***Model 2*** | |
|  | **Crude odds ratio** | **Adjusted odds ratio** |  | **Crude odds ratio** | **Adjusted odds ratio** |
| Surface area (μm^2^) | 1.01 (1.00–1.01) | – | Surface area (μm^2^) | 1.01 (1.00–1.01) | – |
| Age (year) | 1.01 (1.01–1.02) | – | Age (year) | 1.01 (1.00–1.02) | – |
| BMI (kg/m^2^) | 1.03 (1.01–1.05) | – | Smoking history | 1.13 (0.68–1.86) | – |
| SBP (mmHg) | 1.01 (1.00–1.01) | – | Hypertension | 1.42 (1.12–1.81) | 1.22 (1.04–1.43) |
| HbA1c (%) | 1.09 (1.06–1.13) | 1.10 (1.05–1.16) | Obesity ( ≥25 kg/m^2^) | 1.15 (0.89–1.49) | – |
| LDL-c (mg/dL) | 1.00 (1.00–1.00) | – | Dyslipidemia | 1.70 (1.36–2.13) | 1.76 (1.50–2.05) |
| Urinary PCR (mg/g)^*^ | 1.24 (1.14–1.35) | 1.13 (1.01–1.25) | Chronic kidney disease | 1.33 (1.04–1.71) | – |
| Data are expressed as adjusted odds ratios with 95% confidence intervals. Multivariable logistic regression analyses were performed by backward elimination method. *Model 1* was tested in all participants. *Model 2* was tested in diabetic patients. RBC-MF, red blood cell-membrane fluctuation; SBP, systolic blood pressure; LDL-c, low-density lipoprotein cholesterol; PCR, protein-to-creatinine ratio; BMI, body mass index.  * Logarithmic-transformed value was used. | | | | | |

# References

1 Barer, R. Determination of dry mass, thickness, solid and water concentration in living cells. *Nature* **172**, 1097-1098, doi:10.1038/1721097a0 (1953).

2 Lee, S. *et al.* Refractive index tomograms and dynamic membrane fluctuations of red blood cells from patients with diabetes mellitus. *Sci Rep* **7**, 1039, doi:10.1038/s41598-017-01036-4 (2017).

3 M, S. A. *et al.* Impact of Diabetes Mellitus on Human Erythrocytes: Atomic Force Microscopy and Spectral Investigations. *Int J Environ Res Public Health* **15**, doi:10.3390/ijerph15112368 (2018).

4 Guizouarn, H. & Allegrini, B. Erythroid glucose transport in health and disease. *Pflugers Arch* **472**, 1371-1383, doi:10.1007/s00424-020-02406-0 (2020).

5 Lemos, G. S., Marquez-Bernardes, L. F., Arvelos, L. R., Paraiso, L. F. & Penha-Silva, N. Influence of glucose concentration on the membrane stability of human erythrocytes. *Cell Biochem Biophys* **61**, 531-537, doi:10.1007/s12013-011-9235-z (2011).
